# Osteopontin Enhances the Expression and Activity of MMP-2 via the SDF-1/CXCR4 Axis in Hepatocellular Carcinoma Cell Lines

**DOI:** 10.1371/journal.pone.0023831

**Published:** 2011-08-31

**Authors:** Rihua Zhang, Xiaolin Pan, Zuhu Huang, Georg F. Weber, Guoxin Zhang

**Affiliations:** 1 Department of Gastroenterology, The First Affiliated Hospital of Nanjing Medical University, Nanjing, China; 2 Department of Infection Diseases, The First Affiliated Hospital of Nanjing Medical University, Nanjing, China; 3 University of Cincinnati Academic Health Center, College of Pharmacy, Cincinnati, Ohio, United States of America; Institut de Génomique Fonctionnelle de Lyon, France

## Abstract

**Background and Aims:**

Osteopontin, SDF-1α, and MMP-2 are important secreted molecules involved in the pathophysiology of human hepatocellular carcinoma (HCC). This study investigates the effect of the SDF-1α/CXCR4 axis on expression and activity of MMP-2 induced by osteopontin.

**Methods:**

The expression of CXCR4, SDF-1α, MMP-2 and their associated cellular signaling cascades, involving Akt and MAP Kinases, were determined by Western blotting. The activities of MMP-2 and MMP-9 were assayed by gel zymography. The role of the osteopontin receptors integrin α_v_β_3_ and CD44v6 was evaluated using neutralizing antibodies. We also established CXCR4-deficient SMMC7721 cell lines by transfection with miRNA-CXCR4 plasmids and determined cell invasion activity in a transwell assay.

**Results:**

In comparison with untreated cells, recombinant human osteopontin (rhOPN) up-regulated CXCR4, SDF-1α, and MMP-2 expression about 5-, 4-, and 6-fold on the protein levels through binding to integrin α_v_β_3_ and CD44v6 in hepatocellular carcinoma cells (SMMC7721 and HepG2). Inhibition of the SDF-1α/CXCR4 axis down-regulated the rhOPN-induced MMP-2 expression and activity. rhOPN also activated Akt, p38 and JNK. Down-regulation of CXCR4 decreased the rhOPN-induced invasion in SMMC7721 cells.

**Conclusion:**

These results indicate that rhOPN up-regulates MMP-2 through the SDF-1α/CXCR4 axis, mediated by binding to integrin α_v_β_3_ and CD44v6 and activating the PI-3K/Akt and JNK pathways in HepG2 and SMMC7721 cells. Therefore, the osteopontin-SDF-1α/CXCR4-MMP-2 system may be a new therapeutic target for treating HCC progression.

## Introduction

Many experimental and clinical studies have demonstrated that a substantial number of secreted factors are involved in the pathophysiology of human hepatocellular carcinoma (HCC) [1]. Among them, the cytokine osteopontin, the SDF-1α/CXCR4 axis (stromal cell derived factor-1/ CXC chemokine receptor 4), and MMP enzymes are thought to play key roles in invasion and angiogenesis [2], [3], [4].

Osteopontin is an aspartate-rich protein expressed by various tissues and cell types. The existence of variant forms of osteopontin, representing a secreted (sOPN) and intracellular (iOPN) protein, has been described. sOPN interacts with integrins and variant CD44. It contains several cell binding domains, including an arginine-glycine-aspartate (RGD)-motif that engages a subset of cell surface integrins (α_v_β_3_, α_v_β_1_, α_v_β_5_, and α_8_β_1_), a serine-valine-valine-tyrosine-glutamate-leucine-arginine (SVVYGLR)-containing domain that interacts with other integrins (α_9_β_1_, α_4_β_1_ and α_4_β_7_), and an ELVTDFTDLPAT domain that has been reported to bind to integrin α_4_β_1_ [5]. The CD44-binding site has been mapped to the C-terminal portion of osteopontin. The cytokine activates various signaling pathways to mediate multiple functions such as inflammation, cell adhesion, migration and tumor invasion. Osteopontin up-regulates matrix metalloproteinase 2 (MMP-2). In MDA-MB-231 human breast cancer cells, MMP-2 was significantly decreased following exposure to an inhibitor of osteopontin [6]. Further study has shown that osteopontin activates the phosphoinositide 3-kinase/Akt survival pathway [7], [8].

SDF-1 and its receptors, such as CXC chemokine receptor 4 (CXCR4), are thought to play critical roles in motility, homing, and proliferation of many cancer cells [9]. SDF-1, which belongs to the CXC chemokine subfamily, is produced in two forms, SDF-1α (CXCL12α) and SDF-1β (CXCL12β), by alternative splicing of the SDF-1 gene. The binding of SDF-1α to its receptor CXCR4 stimulates receptor dimerization and activates downstream signaling to play an important role in a wide array of disease processes [10], [11], [12], [13].

We thus assessed the role of the SDF-1α/CXCR4 axis in the process of OPN mediated MMP-2 up-regulation in the two human hepatocellular carcinoma cell lines, HepG2 and SMMC7721.

## Materials and Methods

### Materials

rhOPN (Recombinant human Osteopontin/his) (#1433-OP/CF) was purchased from R&D Systems (USA). PD98059 (#9900), LY294002 (#9901), MAPK Family Antibody Sampler Kit (#9926), Phospho-Akt (Ser473), Antibody (#9271) and SDF-1 antibody (#3530) were purchased from Cell Signaling Technology (USA). Rabbit polyclonal to CXCR4 (#ab2074) was obtained from Abcam (USA). Anti-CD44var (v6) monoclonal antibody (#MAB4073), Anti-integrin α_V_ clone AV1 monoclonal antibody (#MAB2021Z) and Rabbit anti-human stromal cell-derived factor-1α affinity purified polyclonal antibody (#AB1868P) came from Millipore (USA). SB203580 (#S8307), SP600125 (#S5567) and ECM gel (#e1270) were obtained from Sigma-Aldrich (USA). AMD3100 (#10011332) was purchased from Cayman Chemical and Functional Grade Purified anti–human CXCR4 (12G5) (#16-9999) from eBioscience (USA).

### Cell culture

The human hepatocellular carcinoma cell lines SMMC7721 and HepG2 cells [14] were cultured in DMEM supplemented with 10% fetal bovine serum (FBS), penicillin (100 U/ml), streptomycin sulfate (100 µg/ml), and maintained at 37°C with 5% CO_2_ in a humid incubator.

### Construction of miRNA-CXCR4 expression plasmids and stable clone selection

Four distinct domains within the coding region of the human CXCR4 cDNA were targeted for RNA interference. For this purpose, four pairs of reverse complementary oligonucleotides were designed and synthesized as Table 1.

**Table 1:** Reverse complementary oligonucleotides.

oligo	5′to 3′
MR079-1-F	TGCTGTAGTAAGGCAGCCAACAGGCGGTTTTGGCCACTGACTGACCGCCTGTTCTGCCTTACTA
MR079-1-R	CCTGTAGTAAGGCAGAACAGGCGGTCAGTCAGTGGCCAAAACCGCCTGTTGGCTGCCTTACTAC
MR079-2-F	TGCTGAACAGTGGAAGAAAGCTAGGGGTTTTGGCCACTGACTGACCCCTAGCTCTTCCACTGTT
MR079-2-R	CCTGAACAGTGGAAGAGCTAGGGGTCAGTCAGTGGCCAAAACCCCTAGCTTTCTTCCACTGTTC
MR079-3-F	TGCTGAACACAACCACCCACAAGTCAGTTTTGGCCACTGACTGACTGACTTGTGTGGTTGTGTT
MR079-3-R	CCTGAACACAACCACACAAGTCAGTCAGTCAGTGGCCAAAACTGACTTGTGGGTGGTTGTGTTC
MR079-4-F	TGCTGATACCAGGCAGGATAAGGCCAGTTTTGGCCACTGACTGACTGGCCTTACTGCCTGGTAT
MR079-4-R	CCTGATACCAGGCAGTAAGGCCAGTCAGTCAGTGGCCAAAACTGGCCTTATCCTGCCTGGTATC
Negative-F	TGCTGAAATGTACTGCGCGTGGAGACGTTTTGGCCACTGACTGACGTCTCCACGCAGTACATTT
Negative-R	CCTGAAATGTACTGCGTGGAGACGTCAGTCAGTGGCCAAAACGTCTCCACGCGCAGTACATTTC

The oligonucleotides were annealed and inserted into the pcDNA6.2-GW/EmGFP-miR expression vector (Invitrogen, #K4936-00) to create pcDNA6.2-GW/EmGFP–miR -CXCR4-1-4, 2-4, 3-1, and 4-4. A control construct was also created.

We used lipofectamine 2000 (Invitrogen, Carlsbad, CA, USA) to separately transfect the five kinds of plasmids into SMMC7721 cells. To select for successful transfectants, the cells were cultured 48 hours after transfection in selection medium containing 3 µg/ml blasticidin (Sigma-Aldrich, Saint Louis, MO, USA). Blasticidin-resistant cells were maintained in culture medium supplemented with 3 µg/ml blasticidin for further analysis.

### Gel zymography for evaluation of gelatinolytic activity

In this study, the human hepatocellular carcinoma cell lines SMMC7721 and HepG2 (1×10^6^) were seeded in 6-cm (diameter) dishes containing complete growth medium. After 12 hours incubation in DMEM with 0.1% BSA, the medium was changed to DMEM with 0.1% BSA in the absence or presence of rhOPN (50 nM) for 60 hours. The rhOPN concentration is in the range commonly associated with cancer [14]. We then collected the supernatant and centrifuged it at 12,000 rpm for 10 min to pellet insoluble material. The protein concentration in the supernatant was determined using a Protein Assay Rapid Kit (Bio-Rad, Osaka, Japan). Samples containing 40 µg total protein in sample buffer (10% SDS, 4% sucrose, 0.25 M Tris-HCl, pH 6.8 and 0.1% bromophenol blue) were used in gelatin zymography. The samples, diluted 1∶1 with 2× sample buffer, were not boiled but warmed in a water bath (55°C) for 3–5 min before being subjected to electrophoresis in a 10% SDS-polyacrylamide gel (SDS-PAGE) containing 0.1% gelatin under non-reducing conditions. The gel was washed twice for 30 min in 2.5% Triton X-100 at room temperature to remove the SDS. After the second wash, all but 2–3 ml of the Triton X-100 was removed, and 100 ml of development buffer (0.05 M Tris-HCl pH 8.8, 5 mM CaCl_2_, 0.02% NaN_3_, 0.02% Brij) was added for further incubation for 24 hours at 37°C. The gel was then stained for 3 hours in Coomassie blue (0.1% Coomassie brilliant blue R250 (w/v) in fixing/destaining solution) and destained in fixing/destining solution (methanol∶ acetic acid∶ water, 4.5∶1∶4.5) until clear bands of gelatinolysis appeared on a dark background. Total activity was analyzed using a scanning densitometer with molecular analysis software (Bio-Rad) [15].

### SDF-1 ELISA

Enzyme-linked immunosorbent assay (ELISA) was done with a human SDF-1 Quantikine kit (R&D), used in accordance with the manufacturer's protocol. In this study, the human hepatocellular carcinoma cell lines SMMC7721 and HepG2 (1×10^6^) were seeded in 6-cm (diameter) dishes containing complete growth medium. After 12 hours incubation in DMEM with 0.1% BSA, the medium was changed to DMEM with 0.1% BSA in the absence or presence of rhOPN (50 nM) for 24, 48, 72 hours. We then collected the supernatants and measured total protein content using the BCA protein assay kit (Pierce) before analysis. Results are representative of three independent experiments.

### Western blotting analysis

The SMMC7721 and HepG2 cells (1×10^6^) were treated with rhOPN (50 nM) for 48 hours, and then lysed in RIPA buffer. Equal amounts of protein (60 µg) were electrophoresed on 12% SDS-PAGE gels and electrophoretically transferred to Immobilon-P membranes (Millipore, Bedford, MA, USA). The membranes were probed overnight at 4°C with antibody to CXCR4 (1∶1000), MMP-2, SDF-1α and monoclonal anti-α-tubulin (1∶5000) in TBST containing 1% BSA (w/v). The blots were then incubated for 2 hours with anti-rabbit or anti-mouse secondary antibodies, the immune complex was detected using an ECL plus detection kit (Pierce, Rockford, IL, USA), and analyzed using a scanning densitometer with molecular analysis software (Bio-Rad).

### Integrin α_v_β_3_ and CD44v6 neutralization

SMMC7721 and HepG2 cells were cultured as described above in the presence of anti-integrin α_v_β_3_ or anti-CD44v6 neutralizing antibodies, or of control IgG. After 60 hours, the cells were collected and Western blotting was performed for the relevant signaling molecules.

### Cell invasion assay

Cell invasion was studied using 24-well transwell plates (Corning Costar, Schiphol-Rijk, Netherland). 60 µl of the ECM gel solution was added to the top compartment of each cell culture insert and dried overnight under laminar air flow. The cells under study were harvested, washed twice with PBS, resuspended in serum-free culture medium with 0.2% BSA and adjusted to a final concentration of 10^6^ per ml. 600 µl serum-free DMEM/ 0.2% BSA containing rhOPN (50 nM) was added to the lower compartment of each well, and 200 µl of the cell suspension was added to the pre-coated upper compartment. The plate with inserts was incubated for 48 hours in a cell culture incubator at 37°C and 5% CO_2_. To determine the background migration, some wells of the 24-well plate were prepared without rhOPN in the lower compartment. Cells remaining on the top side of the filter were removed by soft mechanical dislodging, and the number of cells migrating to the bottom of the filter was counted using a light microscope (in each chamber, six fields were counted at 200× magnification for each condition).

### Flow cytometry

SMMC7721 and HepG2 cells were collected with trypsin/EDTA, washed with fluorescence-activated cell sorting (FACS) buffer (phosphate-buffered saline [PBS], 2 mM EDTA, and 0.5% BSA), and then incubated in FACS buffer for 1 hour at 4°C in the presence of monoclonal antibodies at the manufacturer's recommended concentrations. Binding of anti-CD44var (v6) and anti-integrin α_V_ clone AV1 were visualized with FITC-conjugated rabbit anti-mouse immunoglobulin (Chemicon, Temecula, CA). The cells were washed, fixed with 1% paraformaldehyde and the fluorescence was quantified on 10,000 cells using a FacsCalibur with Cellquest software (BD Biosciences, PharMingen).

### Statistical analysis

The data were analyzed by two-tailed Student's t-test for single comparisons and by one-way analysis of variance for multiple group comparisons. Differences were considered significant at a probability of error below 5% versus control.

## Results

### Osteopontin up-regulates SDF-1α, CXCR4 and MMP-2 expression in hepatocellular carcinoma cells

**Figure 1:**
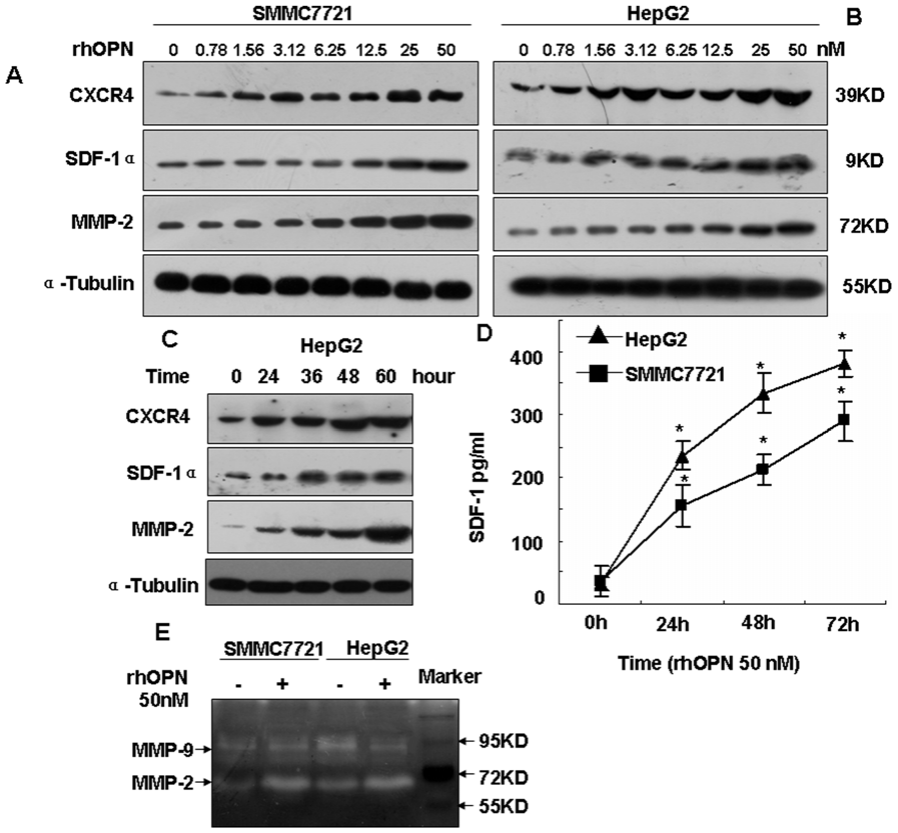
SDF-1α, CXCR4 and MMP-2 expression are induced by rhOPN in SMMC7721 and HepG2 cells. SMMC7721 cells (**A**) or HepG2 cells (**B**) were stimulated with various concentrations of rhOPN for 48 hours, the cells were collected, and SDF-1α, CXCR4 and MMP-2 were detected by Western blotting assay. (**C**) HepG2 cells were stimulated with 50 nM rhOPN for increasing time frames, the cells were collected, and SDF-1α, CXCR4 and MMP-2 were detected by Western blotting assay. (**D**) SDF-1 ELISA of culture supernatants (SMMC7721 and HepG2) after 0–72 hours of rhOPN (50 nM). (E) MMP-2 activity was analyzed by gelatin zymography after stimulation with 50 nM rhOPN for 60 hours in the SMMC7721 and HepG2 cell lines. *denotes *P*<0.05 versus control. The results presented are representative of at least three independent experiments.

To determine the effect of rhOPN on the SDF-1α/CXCR4 axis and MMP-2 expression, Western blotting analysis and gel zymography were done in two human hepatocellular carcinoma cell lines, SMMC7721 and HepG2. Figure 1A shows that the expression of SDF-1α, CXCR4, and MMP-2 protein were induced by rhOPN. There was an apparent increase in the CXCR4 protein level when the concentration of rhOPN was 3.12 nM in SMMC7721 cells, and the same phenomenon was also observed in HepG2 cells (Figure 1B). Figure 1C shows that MMP-2 expression was detectable within 24 hours after the addition of rhOPN, reached a maximum around 60 hours. The MMP-2 levels increased in a time-dependent manner in HepG2 cells. SDF-1α and CXCR4 expression increased accordingly (Figure 1C and 1D). Figure S1A and S1B are quantification of expression described in Figure 1A and 1B based on grayscale analysis.

After identifying MMP-2 protein expression in SMMC7721 and HepG2 cells, we further analyzed the MMP-2 activity in the two cell lines by gelatin zymography. The results demonstrated that the activity of MMP-2 but not MMP-9 was induced by rhOPN at a dose of 50 nM (Figure 1E).

### The SDF-1α/CXCR4 axis is involved in osteopontin-induced MMP-2 expression and activity

**Figure 2:**
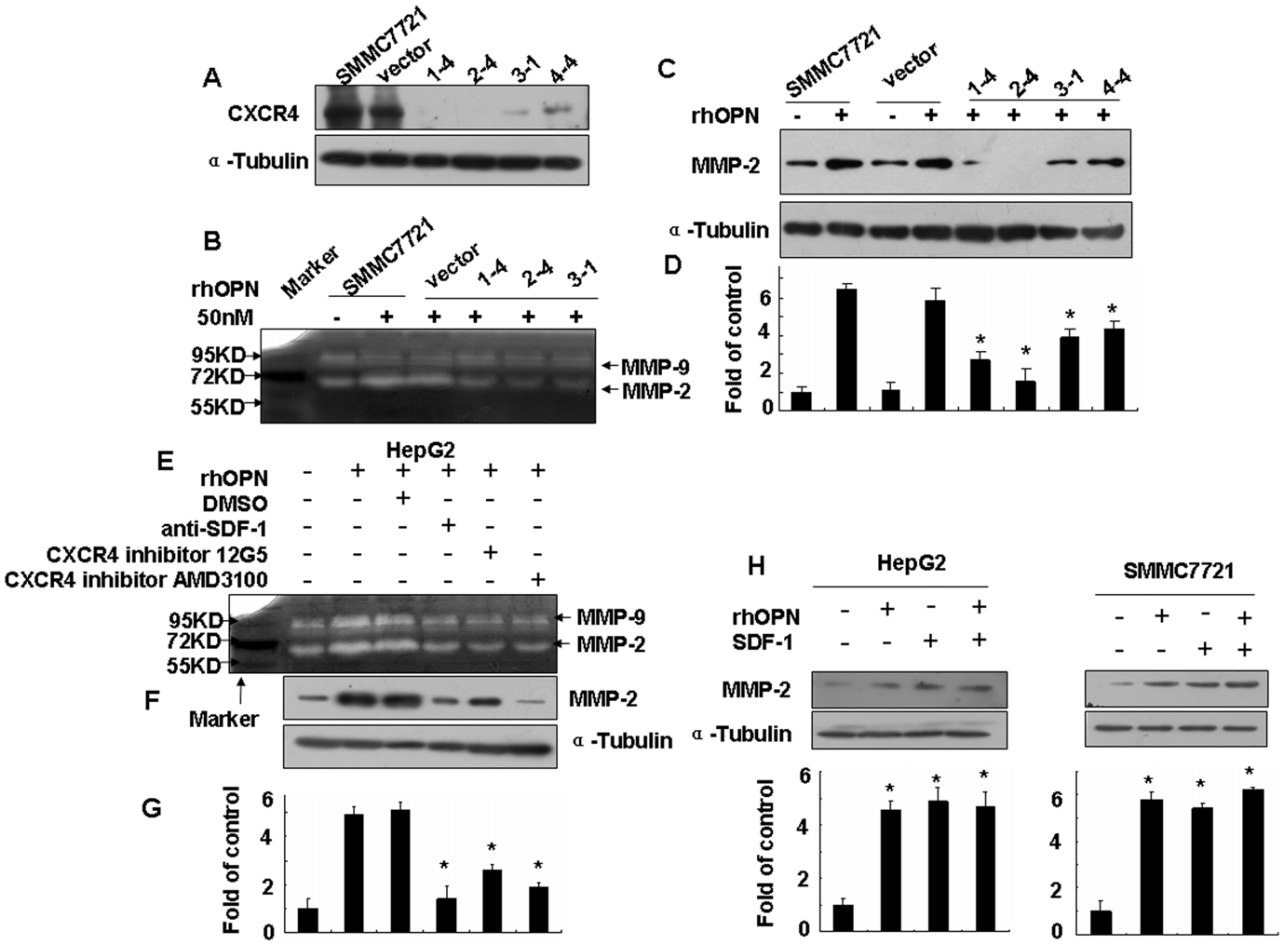
Effects of the SDF-1α/CXCR4 axis on rhOPN-induced MMP-2 expression and activity. (**A**) Verification by Western blotting of the miRNA knockdown of CXCR4 showed a significant reduction of the CXCR4 protein in all clones (1-4, 2-4, 3-1, 4-4). After blocking the SDF-1α/CXCR4 axis with miRNA-CXCR4 and inhibitors (SDF-1 neutralizing antibody at 100 ng/ml, CXCR4 inhibitor 12G5 at 50 µg/ml, or CXCR4 inhibitor AMD3100 at 500 ng/ml), the cells were stimulated by rhOPN in serum-free medium for 60 hours, the cells were collected and analyzed by Western blotting in SMMC7721 cells (**C**) and in HepG2 cells (**F**). The supernatants of SMMC7721 cells (**B**) and HepG2 cells (**E**) were analyzed by gelatin zymography. (**D**) and (**G**) show the densitometric ratio of MMP-2 protein/α-tubulin. (**H**) Western blotting was used to assay the MMP-2 expression induced by rhOPN (50 nM) or/and SDF-1 (30 nM) for 48 hours. * denotes *P*<0.05 versus control. The results presented are representatives of at least three independent experiments.

The MMPs are a large family of proteolytic enzymes, which play an important role in cancer invasion and metastasis due to their ability to degrade the extracellular matrix and basement membrane. Among them, MMP-9 and MMP-2 have been found to be highly associated with metastatic spread by various cancers. Therefore, to determine whether the SDF-1α/CXCR4 axis mediates osteopontin-induced MMP-2 expression and activity, we established CXCR4-deficient SMMC7721 cell lines (clone 1-4, 2-4, 3-1 and 4-4) through the transfection of miRNA-CXCR4. SMMC7721-vector was used as a control. The CXCR4 protein was detected by Western blotting. CXCR4 expression was significantly down-regulated in all of the four miRNA clones (Figure 2A).

The SMMC7721 cells and the miRNA transfectant clones were stimulated with rhOPN for 60 hours. At that time, the cells and their conditioned medium were collected for gelatin zymography and Western blotting. Decreased amounts of MMP-2 proteins were detected in CXCR4-deficient SMMC7721 cells (clones 1-4, 2-4, 3-1 and 4-4) (Figure 2C), compared to SMMC7721 and vector control. The increased activity of MMP-2 but not MMP-9 was abolished in the CXCR4-deficient SMMC7721 cells (Figure 2B).

To further elucidate the role of the SDF-1α/CXCR4 axis in human hepatocellular carcinoma, we detected the expression and activity of MMP-2 induced by 50 nM rhOPN in the presence or absence of SDF-1α neutralizing antibody, CXCR4 inhibitor 12G5, or CXCR4 inhibitor AMD3100. Firstly, we have assessed the toxicity of AMD3100, and the results showed that AMD3100 (500 ng/ml) had no effect on proliferation of HepG2 (figure S2A). The results indicated that exposure of the HepG2 cells to anti-SDF-1 antibody, CXCR4 inhibitor 12G5, or CXCR4 inhibitor AMD3100 decreased the rhOPN-induced MMP-2 activity (Figure 2E) and expression (Figure 2F and D). In order to confirm that SDF-1 actually mediates the observed OPN effects on MMP-2 expression, Western blotting was used to assay the MMP-2 expression levels induced by rhOPN (50 nM) or/and SDF-1 (30 nM) for 48 hours. The results indicate that SDF-1 does generate the same response as OPN in terms of MMP-2 expression, but rhOPN and SDF-1 have no synergistic effects on MMP-2 expression (Figure 2H), presumably because they belong to the same pathway.

### Osteopontin up-regulates CXCR4 in hepatocellular carcinoma cells through both major receptors

**Figure 3:**
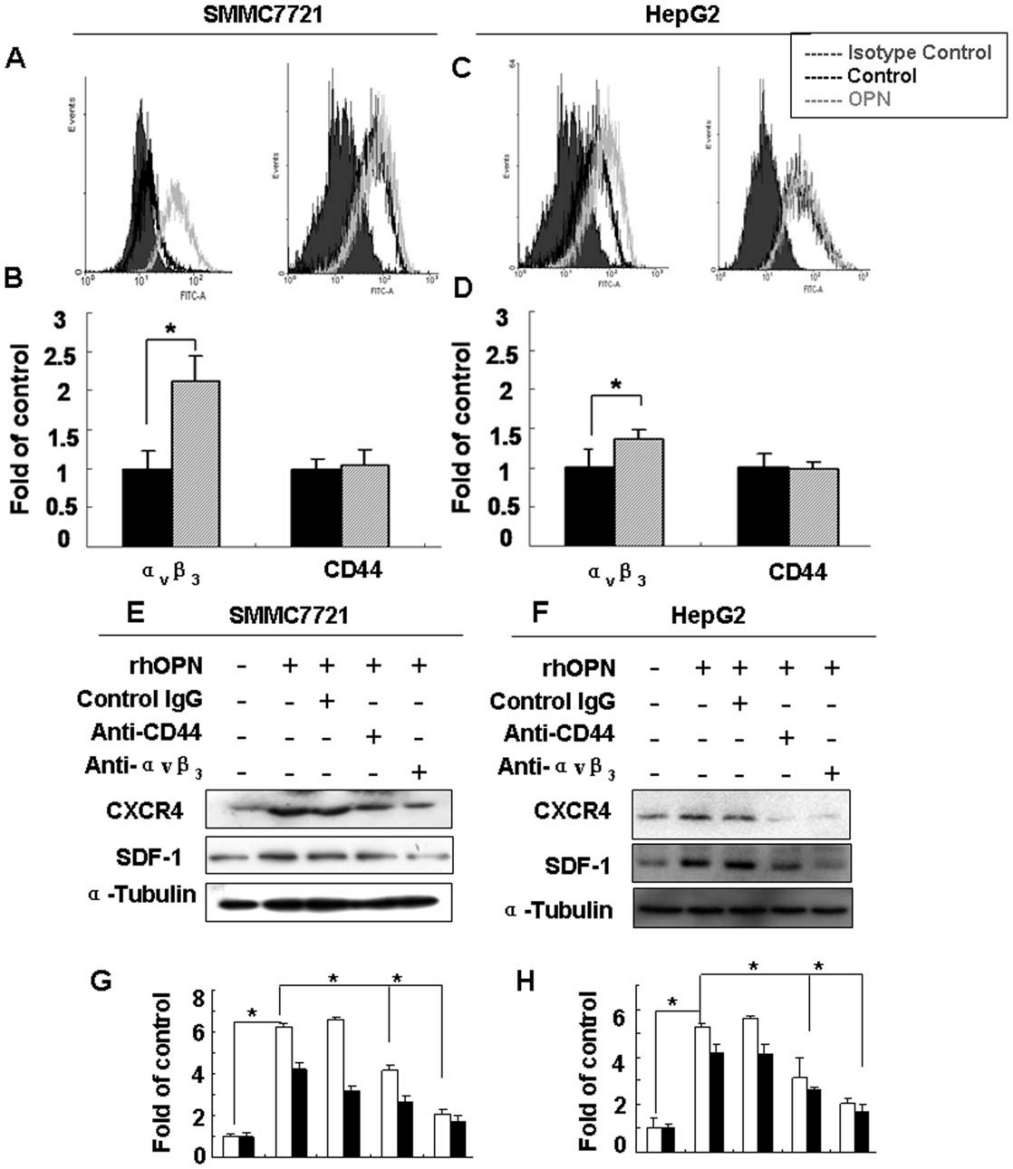
Integrin α_v_β_3_ and CD44 mediated OPN-induced CXCR4 expression in SMMC7721 and HepG2 cells. FACS analysis using monoclonal antibodies to α_v_β_3_ integrin (*left*) and CD44 (*right*) was done for SMMC7721 cells (**A**) and HepG2 cells (**C**), stimulated by rhOPN for 24 hours. The grey area represents isotype control, while the dark line represents the control and the grey line represents the experimental group. SMMC7721 (**E**) and HepG2 (**F**) cells were treated with rhOPN (50 nM), in the presence of neutralizing antibodies to integrin α_v_β_3_ or CD44v6, or control IgG. After 60 hours, the cells were collected and Western blotting was performed to detect CXCR4. (**B**), (**D**), (**G**) and (**H**) are quantitative evaluations. The results are shown as mean ± standard deviation (n = 3). * denotes *P*<0.05 compared to rhOPN treatment in the absence of antibody.

Known receptors for osteopontin through which the cytokine is thought to influence diverse physiological and pathological processes include integrins, most prominently α_v_β_3_, and CD44v3-6. To assess the respective roles of the cognate receptors in osteopontin-dependent signaling pathways, we initially performed flow cytometry analysis in SMMC7721 and HepG2 cells for the expression levels of α_v_β_3_ integrin and CD44. CD44 was expressed a high levels in both cell lines, regardless of whether they had been cultured in the presence or absence of rhOPN. Integrin α_v_β_3_ was about twofold inducible by rhOPN from very low baseline expression levels in SMMC7721 cells (Figure 3A and B). The receptor was only marginally inducible by rhOPN in HepG2 cells (Figure 3C and D).

Neutralizing antibodies to integrin α_v_β_3_ and CD44v6 were used to further test whether these osteopontin receptors are involved in the observed induction of CXCR4 expression. Both antibodies down-regulated the CXCR4 expression induced by rhOPN, about 4.2- and 1.8-fold in SMMC7721 cell (Figure 3E and G), 3.2- and 2.2- fold in HepG2 cell (Figure 3F and H). It is known that CD44 and integrin β_3_ can interact. The contribution by both receptors suggests that rhOPN may engage a CD44v6/integrin α_v_β_3_ complex in the cancer cell membrane, activating downstream signal transduction pathways in the hepatocellular carcinoma cells HepG2 and SMMC7721.

### Osteopontin-induced CXCR4 and MMP-2 expression are mediated by PI-3K/Akt and JNK

**Figure 4:**
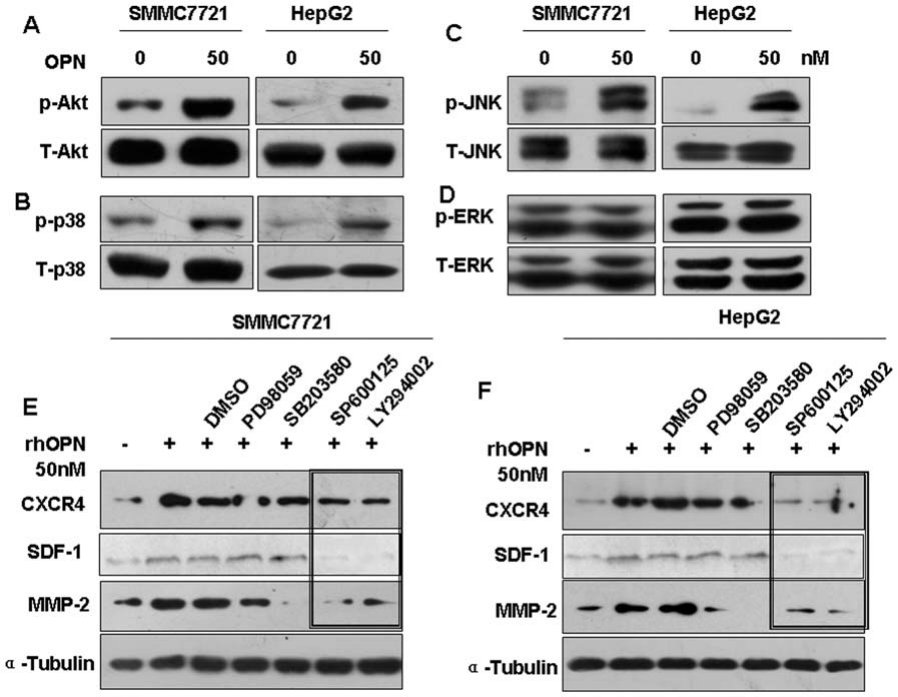
The rhOPN-induced expression of CXCR4 and MMP-2 depends on the PI3K/Akt and JNK pathways. rhOPN induced the phosphorylation of Akt (**A**), p38 (**B**) and JNK (**C**), but not ERK1/2 (**D**) in SMMC7721 and HepG2 cells. The cells (1×10^6^ cells/ml) were left untreated or stimulated with rhOPN (50 nM) for 30 min and total cell lysates were subjected to Western blotting analysis. After pretreatment of SMMC7721 cells (**E**) or HepG2 cells (**F**) with PD98059 (ERK inhibitor, 100 µM), SB203680 (p38 inhibitor, 100 µM), SP600125 (JNK inhibitor, 100 µM), LY294002 (PI-3K inhibitor, 100 µM) or DMSO for 45 min, the cells were treated with rhOPN (50 nM) for 48 hours and total cell lysates were subjected to Western blotting analysis for MMP-2 or CXCR4.

Osteopontin has been reported to activate various kinases such as PI-3K, protein kinase C, and the MAP Kinases, which have three major subgroups (ERK, p38, and JNK). We first asked whether osteopontin activates Akt (the downstream target of PI-3K) and MAPK in SMMC7721 and HepG2 cells. As shown in Figure 4A–D, Akt, p38, and JNK phosphorylation were stimulated within 30 min following the addition of rhOPN, whereas ERK1/2 was not. Figure S3A, B, C and D are quantification of expression described in Figure 4A, B, C, and D based on grayscale analysis (analyzed from three independent experiments, *P<0.05 versus control. The data are representative of three experiments).

To define the role of the PI-3K/Akt and MAPK pathways in rhOPN-induced SDF-1, CXCR4 and MMP-2 expression, we used inhibitors for PI-3K and MAPKs. SDF-1, CXCR4 and MMP-2 expression, stimulated by rhOPN, were decreased significantly in the presence of LY294002 (PI-3K inhibitor) or SP600125 (JNK inhibitor). However, as shown in Figure 4E and F (figure S3E and F are quantification of expression described in Figure 4E and F based on grayscale analysis), the inhibitors of ERK or p38 had little effect on the rhOPN-induced CXCR4 expression in SMMC7721 and HepG2 cells. These results indicate that osteopontin-mediated CXCR4 and MMP-2 expression depends on activation of the PI-3K/Akt and JNK pathways.

### CXCR4 is required for osteopontin-induced cell invasion

**Figure 5:**
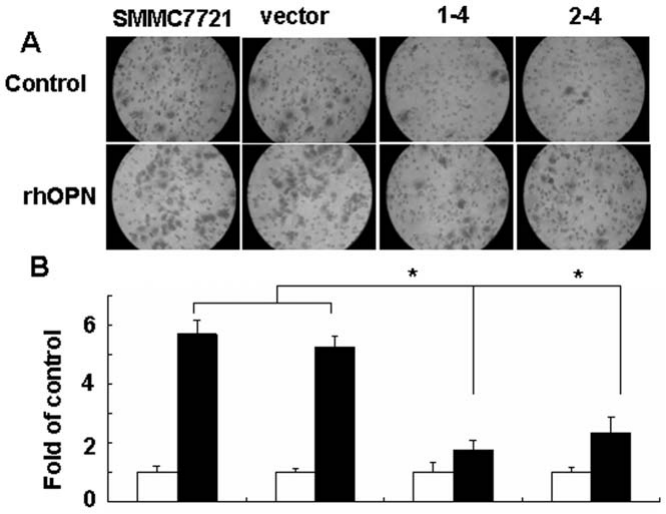
The rhOPN-induced SMMC7721 cells invasion is mediated by CXCR4. The invasion assay was set up in transwell chambers. Cell culture inserts with 8.0-µm pore diameter were used to separate the top and bottom chambers. 60 µl of ECM gel solution was added to the upper compartment of each cell culture insert and dried overnight under laminar air flow. SMMC7721 parent cells, vector controls, and CXCR4 miRNA clones (1-4, 2-4) were plated onto the membrane of the top chamber, and rhOPN was administered to the bottom chamber. After 48 hours, the cells that had invaded to the lower surface of the membrane were enumerated. (**A**) Bright-field image of cells migrated to the bottom of chambers on the inserts (200× original magnification). (**B**) Quantification of cell invasion. The open bars represent no osteopontin, the filled bars represent rhOPN treatment. In each chamber, six fields were counted at 200× magnification for each condition by two investigators. * indicates *P*<0.05 versus control. The data are representative of three experiments.

The observation that rhOPN induced SDF-1α, CXCR4 and MMP-2 expression in human hepatocellular carcinoma cells suggested that the SDF-1α/CXCR4 axis might play a role in osteopontin-dependent tumor progression. Therefore, we assessed the invasive response to rhOPN in a transwell assay. As shown in Figure 5, rhOPN stimulated the invasion response of SMMC7721 cells (6-fold) and SMMC7721-miRNA-CXCR4 cells, clones 1-4 and 2-4 (2-fold). Importantly, invasiveness was decreased in clone 1-4 and 2-4 cells, about four fold lower than in SMMC7721cells. These results indicate that rhOPN activates cell invasion through the induction of CXCR4 in SMMC7721 cells.

## Discussion

Hepatocellular carcinoma (HCC) is one of the most common and malignant neoplasms worldwide. Its pathophysiology is associated with multiple cytokines and secreted factors, including osteopontin, SDF-1 and its receptor CXCR4, as well as MMP-2 and MMP-9 [16], [17], [18]. Osteopontin expression is up-regulated in tumors and blood of human HCC patients compared to healthy controls [19], [20], [21]. It has been suggested that osteopontin overproduced by tumor cells may act as a potent angiogenic factor [22]. Our study indicates that osteopontin stimulates MMP-2 expression and activity through a hitherto undefined pathway.

**Figure 6:**
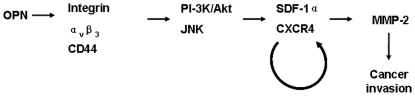
Model for the mechanism of osteopontin-dependent MMP-2 up-regulation in hepatocellular carcinoma. The results of the present study show that osteopontin up-regulates SDF-1α, CXCR4, and MMP-2 via integrin α_v_β_3_ and CD44v6, as well as PI-3K/Akt and JNK. These data are consistent with an osteopontin-induced autocrine loop of SDF-1α/CXCR4 activation that leads to tumor invasion, in part via MMP-2 secretion.

Both MMP-2 and MMP-9 play important roles in the pathogenesis of many cancers [19], [20]. Our results are consistent with previous results showing that osteopontin up-regulates MMPs [21], [22], [23]. It has been reported that the osteopontin-induced activation of MMP-2 or MMP-9 is mediated by the PI-3K/Akt/NF-κB signaling pathway [21], [24], [25]. Osteopontin may promote the activation of pro-MMP-9, but not MMP-2, through an NADPH oxidase-associated signaling cascade [22]. While we found no rhOPN effect on MMP-9, our study has identified a novel pathway to MMP-2 expression and activation, which is mediated by the SDF-1α/CXCR4 axis (Figure 6). Our results (Figures 4) further demonstrate that the p38 and ERK pathways are involved in the expression of MMP-2, but not SDF-1 and CXCR4 expression, induced by rOPN. In this study, our focus is on the OPN-dependent enhancement of the expression and activity of MMP-2 via the SDF-1/CXCR4 axis. p38 and ERK MAPK induces MMP-2 expression in many cells, for example, baicalein downregulates the protein expression levels of MMP-2 by inhibiting the expression of p-Akt, p-ERK, p-p38 and p-JNK [26]; the down-regulation of p38 MAPK and JNK by siRNA transfection resulted in a decrease in MMP-2 expression by MelJuso cells [27]. The rhOPN-induced CXCR4 expression is dependent on CD44 and integrin receptors, and is regulated by the PI-3K/Akt and JNK pathways (Figures 3 and 4) in the two hepatocellular carcinoma cell lines (HepG2 and SMMC7721) tested. Moreover, decreased production of CXCR4 and MMP-2 in association with lower invasion of hepatocellular carcinoma cells could be related to the down-regulation of metastasis [28], [29]. Our data add to this evidence (Figure 5), indicating that osteopontin, CXCR4, and MMP-2 are key cytokines for HCC progression. Although our results are derived from two model cell lines, published evidence corroborates that they are relevant for human cancers [30].

Osteopontin and CXCR4 have been used as markers for immune activation [31] for homing precursor cells [32], [33] and for metastasizing cancer cells [34], [35], [36]. Further, osteopontin and CXCR4 may serve as early biomarkers for cancer detection [37]. However, reports in the literature have not yet provided a functional link between these molecules. The identification of CXCR4 as a downstream target of osteopontin and an essential mediator in the induction of MMP-2 closes this gap. Osteopontin up-regulates MMP-2 through activating the SDF-1α/CXCR4 axis, mediated by binding to integrin α_v_β_3_ and CD44v6 and activating the PI3K/Akt and JNK pathways in hepatocellular carcinoma cells (HepG2 and SMMC7721). Therefore, the osteopontin-SDF-1α/CXCR4- MMP-2 system may be a promising therapeutic target.

## Supporting Information

Figure S1 **Figure S1A and S1B are quantification of expression described in** **Figure 1A and 1B** **based on grayscale analysis (analyzed from three independent experiments).** *denotes *P*<0.05 versus control.
(TIF)

Figure S2 **The cell numbers at 12 h postplating were set as 1, and cell numbers collected at all other time points were compared with the initial values at 12-h time point.** Results were expressed as the mean±SD. * *P*<0.05 when compared with the DMSO control.
(TIF)

Figure S3 **Quantification of expression described in** **Figure 4A, B, C, D, E and F** **based on grayscale analysis (analyzed from three independent experiments).** *P<0.05 versus control. The data are representative of three experiments.
(TIF)
