# Supplementary figures and images for: Osteopontin Enhances the Expression and Activity of MMP-2 via the SDF-1/CXCR4 Axis in Hepatocellular Carcinoma Cell Lines
Source: PLoS One. 2011 Aug 31;6(8):e23831. doi: 10.1371/journal.pone.0023831 (PMC3166084; doi:10.1371/journal.pone.0023831)

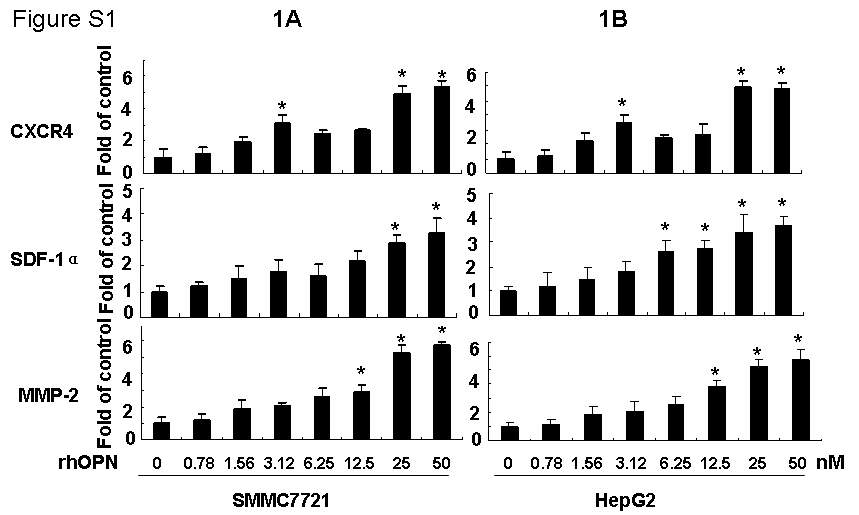

Supplement: Figure S1 — A and S1B are quantification of expression described in Figure 1A and 1B based on grayscale analysis (analyzed from three independent experiments). *denotes P<0.05 versus control. (TIF) [file pone.0023831.s001.tif]

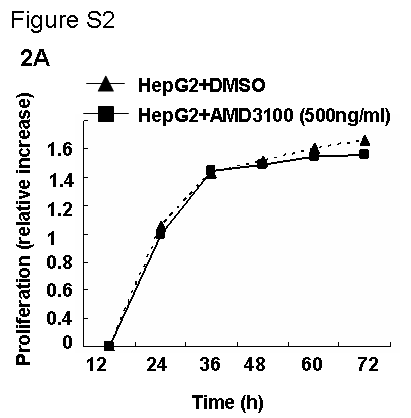

Supplement: Figure S2 — The cell numbers at 12 h postplating were set as 1, and cell numbers collected at all other time points were compared with the initial values at 12-h time point. Results were expressed as the mean±SD. * P<0.05 when compared with the DMSO control. (TIF) [file pone.0023831.s002.tif]

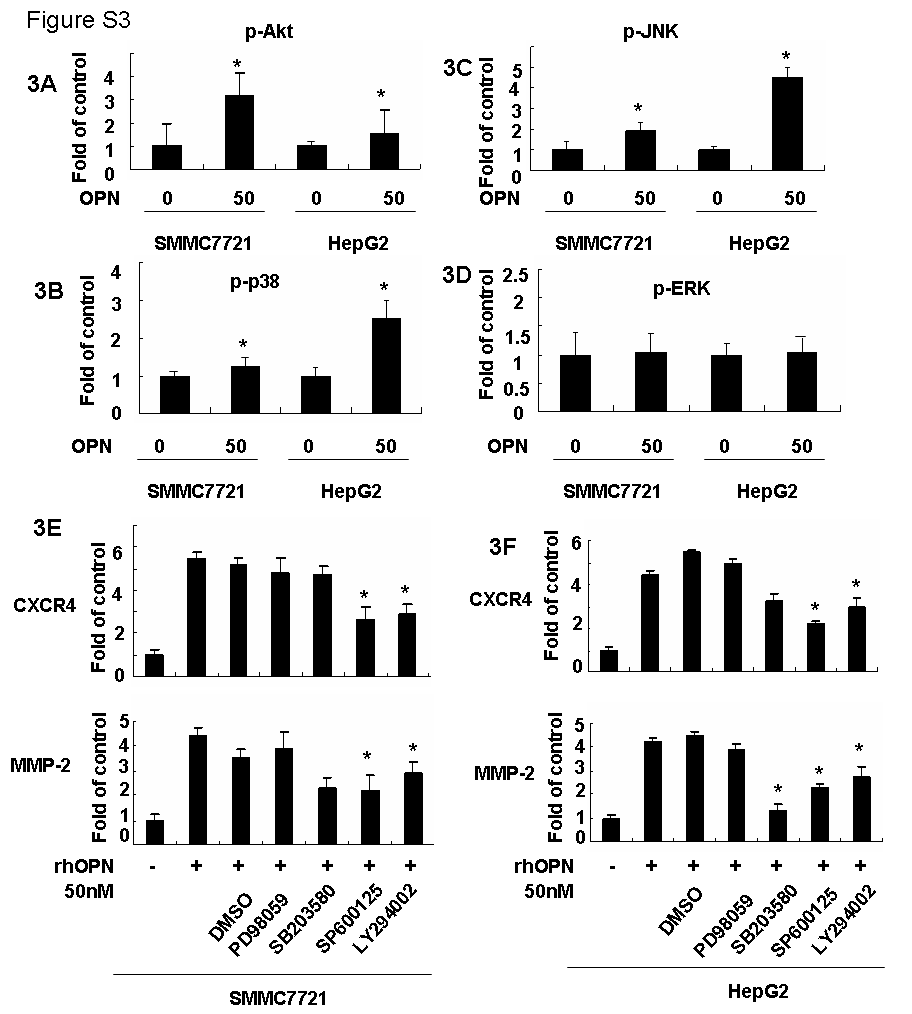

Supplement: Figure S3 — Quantification of expression described in Figure 4A, B, C, D, E and F based on grayscale analysis (analyzed from three independent experiments). *P<0.05 versus control. The data are representative of three experiments. (TIF) [file pone.0023831.s003.tif]
